# The Interpersonal Mindfulness Program for Health Care Professionals: a Feasibility Study

**DOI:** 10.1007/s12671-020-01477-5

**Published:** 2020-08-26

**Authors:** Agna A. Bartels-Velthuis, Erik van den Brink, Frits Koster, H. J. Rogier Hoenders

**Affiliations:** 1 Lentis Psychiatric Institute, Center for Integrative Psychiatry, Hereweg 76, 9725 AG Groningen, the Netherlands; 2 University of Groningen, University Medical Center Groningen, University Center for Psychiatry, Rob Giel Research Center, University of Groningen, Hanzeplein 1, 9713 GZ Groningen, the Netherlands; 3 MBCL Training & Therapie, Koelandsdrift 10, 9755 PN Onnen, the Netherlands; 4 Trainingsbureau Mildheid & Mindfulness, Peperweg 9, 9891 AK Ezinge, the Netherlands

**Keywords:** Mindfulness, Self-compassion, Empathy, Stress, Professional quality of life

## Abstract

**Objectives:**

There are a number of mindfulness-based programs (MBPs) that have demonstrated effectiveness for patients and health care professionals. The Interpersonal Mindfulness Program (IMP) is a relatively new MBP, developed to teach those with prior mindfulness training to deepen their mindful presence, empathy and compassion in the interpersonal domain. The aim of the present study was to examine the feasibility of using the IMP with mental health care workers and assessing its effects on levels of mindfulness, self-compassion, empathy, stress and professional quality of life when compared with the control group participants.

**Methods:**

The IMP training consisted of nine weekly 2.5-h sessions and daily home practice (45–60 min). Twenty-five participants (mean age, 51.4 years) with mindfulness experience participated in the training. Twenty-two individuals in the control group (mean age, 47.5 years) were recruited from those who had followed a mindfulness training before. Feasibility of the IMP was assessed in the training participants in six domains. All study participants completed self-report questionnaires before and after the training.

**Results:**

The IMP training was considered highly acceptable and very useful. The training had a significant positive effect on self-compassion, empathy and compassion fatigue, but no effect on mindfulness, stress and compassion satisfaction. Five participants reported some mild adverse reactions.

**Conclusions:**

The IMP training appears feasible for health care professionals and seems to induce some positive effects. A few mild adverse effects were reported. Further research on the effectiveness and possible mechanisms of change of the IMP training in larger samples is needed.

Mindfulness is now a well-established part of (mental) health care. Most (mental) health care institutions offer mindfulness training to patients for various disorders (Creswell 2017; Veehof et al. 2016; West et al. 2016). The benefits of mindfulness for therapists have been recognised by a number of researchers (Hick and Bien 2008; Surrey and Kramer 2013, Wilson and Dufrene 2008). As health care workers seem to be prone to empathy fatigue already in the training phase, mindfulness-based programs (MBPs) may help prevent empathy fatigue and burnout and contribute positively to self-awareness, self-compassion and empathic ability (Karpowicz et al. 2009; Saunders et al. 2007; Shapiro et al. 2005, 2007). In a study of a large sample of different types of mental health professionals by Rossi et al. (2012), psychiatrists and social workers were shown to have the highest levels of burnout and compassion fatigue.

MBPs invite mindfulness into all moments of one’s life, including interpersonal situations. There is preliminary evidence that mindful presence leads to interpersonal benefits (Parker et al. 2015). Indeed, attentive and careful attunement to oneself and others can have a calming, stress-reducing effect and cause deep resonance on a neurobiological level (Porges 2011; Siegel 2007). It seems likely that mindfulness as a trait benefits work performance and interpersonal relationships (Mesmer-Magnus et al. 2017) and reduces burnout in mental health professionals (Di Benedetto and Swadling 2014). Most of the formal practice in Mindfulness-Based Stress Reduction (MBSR) is individual practice and specific instructions on how to include the interpersonal domain are lacking. Given how easily mindful presence is lost in the more challenging interpersonal situations, it makes sense to first establish sufficient stability in mindfulness through individual practice. Once established, additional benefits are to be expected from formal practice in mindful communication, as proposed by Kramer et al. (2015) in their novel Interpersonal Mindfulness Program (IMP), which arose from collaboration between the University of Massachusetts and Metta Programs (Kramer et al. 2008) and is a secular adaptation of Insight Dialogue (Kramer 2007). The IMP expands on the work started in MBSR by deepening insight into what heals and what harms through guided interpersonal mindfulness practice in pairs, small groups or during plenary exchange. It presents a methodical way supported by clear guidelines and contemplations, directly cultivating mindful presence, empathy and compassion in the interpersonal domain. As empathy fatigue depends on interpersonal experience and skills, the IMP might lead to improvement beyond conventional MBPs.

The aim of this study was to examine the feasibility, acceptability and preliminary effectiveness of the IMP training in line with Bowen et al. (2009). We explored whether the IMP has additional benefits for health care workers who had already followed an MBSR course or equivalent MBP. We hypothesised that after following the IMP, the training participants will report higher levels of mindfulness, self-compassion and empathy, less stress and a higher professional quality of life, compared with a control group.

## Method

### Participants

Participants were recruited mainly from an outpatient clinic serving people with mood and anxiety disorders. Inclusion criteria for participating in the training were: (i) having previously completed a mindfulness training (MBSR or Mindfulness-Based Cognitive Therapy (MBCT)) and (ii) willingness to spend 45–60 min daily on practicing at home in accordance with the course material. Participation in the study was voluntary. The individuals in the control group were recruited from the list of health care professionals who had followed a mindfulness training at our department, in the years 2012–2015. All those who agreed to participate were included in the control group. Our study was qualified as being exempt from review by the Medical Ethics Committee of the University Medical Center Groningen (METc 2017/324, ref. M17.215528 dated 26 July 2017)), based on the regulations of the Central Committee on Research in Human Subjects Act (WMO in Dutch), given the low frequency of the assessments and the psychologically non-probing nature of the questions. Informed consent was obtained from all study participants.

### Procedure

#### Training

The teachers complied with the IMP manual as developed by Kramer et al. (2015). This manual was translated into Dutch by the experienced mindfulness teachers who participated in the first IMP Teacher Training in the Netherlands in 2015 (including two of the authors, EvdB and FK). The manual was translated literally, except for some style adjustments oriented towards the Dutch context and for a number of poems that were replaced by suitable Dutch poems in the same atmosphere.

The training comprises: (1) Practice with the six guidelines of Insight Dialogue (in pairs or larger groups): pause, relax, open, trust emergence, listen deeply and speak the truth, and (2) Exploration in mindful dialogue, supported by the guidelines, of contemplation themes, including pleasant-neutral-unpleasant feeling tone of experiences; experiences of differences, judgments, constructs of inferiority and superiority; giving and receiving; impermanence and change; roles in professional and personal life; desires and hindrances in communication; cultivating what is wholesome. Table 1 presents an overview of the guidelines, themes and contemplations of the sessions and Table 2 depicts an average session.

**Table 1:** Session overview interpersonal mindfulness program

Session (2.5 h)	Guideline	Theme	Contemplations
1	PAUSE	Pleasant-unpleasant experiences	- Attending to internal sensations - Attending to external sensations - Investigating unpleasant and pleasant experiences with people
2	(Pause) RELAX	Differences	- Defining the self - Investigating differences, judgments and constructs of inferiority and superiority - Investigating feelings of differences, separation and judgments
3	(Pause, Relax) OPEN	Giving and receiving	- Giving and receiving - Letting go/letting be
4	(Pause, Relax, Open) TRUST EMERGENCE	Impermanence^a^	- Investigating impermanence in participants’ lives: change across time - Resisting change - Attuning to change in the present moment
5	(Pause, Relax, Open, Trust Emergence) LISTEN DEEPLY, SPEAK THE TRUTH	Roles	- Roles in community, society, work: noticing content, words and phrasing - Roles in intimate relationships and family: noticing meaning conveyed by the body - Roles of self: who am I? Awareness of the present moment - Gratitude
6	ALL	Hungers^b^	- The interpersonal hunger for pleasure and the urge to avoid pain - The interpersonal hunger to exist in the eyes of the other, to be seen and the fear of invisibility - The interpersonal hunger to avoid being and the fear of being seen
7	LISTEN DEEPLY, SPEAK THE TRUTH	Hindrances^c^	- Hindrance of anger and aversion: noticing content, words and phrasing - Hindrance of remorse and worry: noticing meaning conveyed by the body - Hindrance of doubt: awareness of the present moment
8	ALL	Cultivating the wholesome	- Recollecting our virtues
9	Closure, review of all guidelines		- Reflecting on impact of the guidelines in interactions - Noticing impermanence - Anticipating future change

Note: For logistic reasons, the 5th-day session of 7 h as scheduled in the original IMP manual had to be replaced by a session of equal duration as the other sessions. Salient themes and practices were not changed. Source: Kramer et al. (2015)

^a^Impermanence: the human condition of experiencing change in different ways

^b^Hungers: exploration of different types of desire

^c^Hindrances: inner tendencies that can stagnate or obstruct wise and healthy behaviour—limiting habits one may meet in life

**Table 2:** An average session of the IMP training

An average session of the IMP training	
• Arrival meditation: partly in silence, partly guided, the trainer recalls guidelines and themes from earlier sessions. • Check-in with the whole group: participants can share from practicing with the guidelines and themes in the past week. In group sharings, generally no round is made and participants mindfully choose their moment to speak or listen, while practicing with the guidelines. • Introduction of the guideline(s) and themes from the current session. • Practices in pairs or, later on in the course, small groups with 1 or more guidelines and contemplations. This is the main part of the session and often the following structure is used: in a first round, person A is invited to speak and person B to listen. In a second round, A and B switch roles. In a third round, A and B will reflect in mindful dialogue on the experiences as a speaker and as a listener in previous rounds. • As needed, sitting is alternated with mindful walking, moving or lying. A short pause is optional as an opportunity for informal practice. • Group reflection: participants share insights from the practices in pairs. • Home practice assignments and handouts: daily formal individual practice by choice (e.g. 30 min sitting meditation, body scan, yoga and/or walking meditation), reflection on themes from the session concerned and informal practice with the guidelines in interpersonal contacts. • Closing meditation: loving kindness towards oneself and others.	

The training consisted of nine weekly 2.5-h sessions. The IMP training is designed as an eight-session course with a practice day between sessions 4 and 5, but for logistic reasons, the original practice day had to be replaced by a 2.5-h session. The minimum number of participants per training group was set at 10, the maximum at 14. Participants received a training manual (Koster and Van den Brink 2015), a Dutch translation of the Students Home Practice Packet as included in the IMP manual, expanded by summaries of contemplation themes and audio material of guided meditations introducing the six guidelines, which were recorded live in the sessions. Some translatable English poems and texts as suggested in the IMP manual were added to the training manual and some equivalent Dutch ones were added.

#### Teachers

Both teachers are certified by the Dutch Association for Mindfulness-Based Trainers (VMBN, category 1) and have extensive meditation experience (25 and 40 years, respectively). They have been engaged in meditation practices, both in person as in a professional context (teachers/supervisors at the Dutch Institute for Mindfulness, the Institute for Mindfulness-Based Approaches and as leaders of retreats). One of them (EvdB) is a psychiatrist/psychotherapist, the other (FK) a specialised psychiatric nurse and teacher in mindfulness and compassion programs. Both teach mindfulness and compassion in secular Train the trainer institutes throughout Europe.

### Measures

To assess feasibility of the IMP, the training participants completed an evaluation form directly after the last training session. All study participants completed five self-report questionnaires, before and after the training, and their gender and age were assessed.

#### Feasibility

The feasibility of the IMP training was assessed in the domains described by Bowen et al. (2009). *Acceptability* was operationalised as overall program satisfaction, personal and professional benefit and willingness to recommend the program to others. *Demand* was supposed to be present given the prevalence of distress and burn-out in mental health care workers (see the Introduction) as well as given the existence of the IMP developed by Kramer et al. (2015). *Implementation* was operationalised as the extent to which the program could be fully delivered. It was evaluated by the number of cancelled, truncated or postponed sessions as well as participants’ satisfaction with the course material and the training organisation. *Practicality* was evaluated by program attendance and duration of daily home practice. *Adaptation* was operationalised as modifications made to the IMP. *Integration* was operationalised as the extent to which participants used the learned techniques in everyday life and how they plan to do so in the future. The domain *Expansion* was not applicable in this study as—to our knowledge—the IMP is novel and has not yet been scientifically studied. *Preliminary Effectiveness* was operationalised as effect sizes of changes in self-reported levels of mindfulness, self-compassion, empathy, stress and professional quality of life. Moreover, possible adverse reactions to the training were assessed.

#### Mindfulness

The 39-item Five Facet Mindfulness Questionnaire (FFMQ; Baer et al. 2008; Dutch version: Bohlmeijer et al. 2011) assesses five aspects of mindfulness (observing, describing, acting with awareness, non-judging of inner experience and non-reactivity to inner experience), which in general apply to the participant. Each item has to be rated on a 5-point scale (ranging from 1 to 5), higher scores denoting a higher level of mindfulness. Cronbach’s alpha for the FFMQ total score in the current study was excellent (*α* = 0.91).

#### Self-Compassion

The 24-item Dutch version (Neff and Vonk 2009) of the Self-Compassion Scale (SCS; Neff 2003) measures the degree of self-compassion. The SCS has six subscales (self-kindness, self-judgement, common humanity, isolation, mindfulness and over-identification). Each item has to be rated on a 5-point scale (ranging from 1 to 5), higher scores denoting a higher level of self-compassion. Reliability of the SCS total score in this study was excellent (*α* = 0.90).

#### Empathy

Level of empathy is measured with the Empathy Quotient (EQ; Lawrence et al. 2004; Dutch translation Groen et al. 2015), a self-report measure with 40 propositions for each of which the participant has to rate to what extent (s)he agrees, ranging from 0 (totally agree) to 3 (totally disagree). Reliability of the EQ in this study was good (*α* = 0.84).

#### Stress

The ten-item Perceived Stress Scale (PSS; Cohen et al. 1983) measures the degree to which people perceive their lives as stressful in the last month. Reliability of the PSS in this study was good (*α* = 0.87).

#### Professional Quality of Life

The Professional Quality of Life Scale: Compassion Satisfaction and Compassion Fatigue Version 5 (ProQOL; Hudnall Stamm (2009); Dutch translation: Bartels-Velthuis et al. (2016); www.proqol.org) has 30 items about the participant and his/her current work situation, covering the last 30 days. The items are rated on a 5-point scale, ranging from 1 (never) to 5 (very often). The ProQOL has two subscales that were both used in this study: Compassion Satisfaction (10 items), representing the positive aspects of the work as a helper, and Compassion Fatigue (20 items), representing the negative aspects of the work as a helper. Higher scores denote higher levels of satisfaction and fatigue, with Cronbach’s alphas in this study amounting to 0.84 and 0.76, respectively, pointing at a good and acceptable reliability, respectively. The subscale Compassion Fatigue comprises two subscales, the Burnout Scale and the Secondary Trauma Scale, which were not used in the analyses, as the Burnout Scale showed an alpha of 0.57, denoting a poor reliability, and the Secondary Trauma Scale an alpha of 0.69, which is just below an acceptable reliability.

### Data Analyses

Analyses were carried out with the statistical package IBM SPSS, version 25. The Kolmogorov-Wilk test, which has to be used in relatively small (*n* < 50) samples, showed that all continuous variables were normally distributed. Differences in the mean changes in the outcome from pre to post in the two groups were measured directly by the time*group interaction term in a repeated measures ANOVA. Partial eta squared values as a measure of effect sizes were calculated, with values of 0.01, 0.06 and 0.14 being considered as small, medium and large effect sizes, respectively. A two-tailed alpha level of 0.05 was used. Whether the amount of home practice was related to the outcome in the intervention group was also explored. Therefore, a variable (‘minutes home practice per week’) was composed by adding number of minutes of exercise per week and number of minutes studying course material per week. Subsequently, difference scores of the six outcome measures (i.e. post-test scores minus pre-test scores) were calculated. Finally, Pearson correlation coefficients of difference scores and minutes home practice per week were calculated. The teachers were not involved in processing nor in analysing the data.

## Results

Table 3 presents the professional categories to which the training participants and the persons in the control group belong. Twenty-four (of twenty-five) training participants completed the evaluation form. Forty-seven professionals participated in the effectiveness study, of whom twenty-five followed the IMP training and twenty-two were in the control group.

**Table 3:** Professional categories of the study participants

Profession (category)	Training participants (*n*)	Control group (*n*)
Doctor of medicine	3	1
Psychologist	6	6
Nurse	7	9
Other^a^	9	6
Total	25	22

^a^The category ‘Other’ includes professions such as mindfulness teacher, social worker, mental health care counsellor, physiotherapist and drama therapist

### Acceptability

The mean grade given to the total training (on a 0–10 scale) amounted to 8.2 (SD = 0.7). The training was estimated (answer options: not, slightly and very relevant) to be very relevant by 88% and to be slightly relevant by 13% of the participants for one’s own profession. For one’s personal life, the training was very relevant according to 83% and a bit relevant according to 17%. Table 4 shows in detail what the training participants had learned after following the IMP training. In additional comments, several participants emphasised how helpful the guidelines were and how the training brought more awareness and rest in their communication (details not shown). Finally, 87.5% would recommend to follow the training to others and 12.5% would maybe do so.

**Table 4:** What participants had learned from the IMP training

After having followed the training …	Totally disagree (%)	Somewhat disagree (%)	Do not agree/do not disagree (%)	Somewhat agree (%)	Totally agree (%)
I have learned to pause more often when I am talking		4		33	63
I have learned to relax more when communicating with others		4		42	54
I have learned to communicate with others with more openness and less inner preparation			8	33	58
I have learned to tell more precisely and with respect for myself and for the other what is important for me at the moment			13	54	33
I have learned to listen more mindfully		4	13	35	48
I have learned to be aware of physical sensations			8	54	38
I have learned to be aware of my emotions			13	50	38
I have learned to be aware of my thoughts and judgments			17	38	46
I gained more insight into unhealthy habits	4	8	4	58	25
I gained more insight in how I can better take care of myself		9	17	44	30
I can indeed better take care for myself		13	17	58	13
I have more self-confidence	8		13	54	25
I am in a better mood	9		22	52	17
I can better deal with physical pain	8	4	46	29	13
I can better deal with painful emotions	4		39	39	17
I can better deal with unpleasant thoughts	4	9	22	48	17
I am more aware of what is stressful in my life		4	17	46	33
I am more aware of stressful situations at the moment they occur		4	4	71	21
I am more skilled to handle stress	4		9	70	17
I can improve my own health	4	8	17	54	17
the relation with myself has improved	4		8	58	29
the relation with others has improved		4	13	63	21

### Implementation

The training was fully delivered. Satisfaction with the teachers (on a 0–10 scale) was given mean grades of 8.3 (SD, 0.8; range, 7–10) and 8.4 (SD, 0.6; range, 8–10) to EvdB and FK, respectively. Satisfaction with the course material (scored on a 5-point scale, ranging from 1 = very unsatisfied to 5 = very satisfied) amounted to 3.9 (SD = 0.5; range, 3–5). Satisfaction with the training organisation (calculated as the sum of scores on ‘location’ and ‘prior information about the training’, both scored on a 5-point scale) scored a mean grade of 7.4 (SD, 1.2; range, 5–10).

#### Practicality

All training participants completed the IMP training. Mean number of times they had done exercises at home was 3.6 (SD, 2.1; range, 1–8), mean time spent on home practice amounted to 92.4 min (SD, 81.3; range, 5–300) and mean time spent on studying the course material was 28.7 min (SD, 14.7; range, 10–75).

#### Adaptation

In other than the current training sessions, participants informed the teachers that the language of the Dutch IMP manual could be more secular and less Buddhist. Particularly terms like ‘impermanence’ (session 4), ‘hungers’ (session 5) and ‘hindrances’ (session 6) are strongly associated with Buddhism. In the session overview (Table 1), these terms are explained. However, the Dutch IMP manual was not adapted accordingly, apart from replacing the in the Dutch language in this context unfamiliar use of the word hungers by the more accessible word ‘desires’. Some participants found the name of the fourth guideline ‘Trust Emergence’ difficult to grasp. This guideline invites us to meet our ever-changing, impermanent experience with beginner’s mind and surrender to the flow of the communication process. Participants often misunderstood this, as if all experience should be trusted. Meanwhile, the latest version of the IMP manual 2019 has changed this name into ‘Attune to Emergence’, which is less likely to be misunderstood. Also, in the 2019 version, hindrances has been replaced by ‘limiting habits’, which has less strong Buddhist associations.

#### Integration

Of the 24 training participants, 22 responded to the question about their future plans in the field of interpersonal mindfulness. Of these, 17 planned to continue practicing and applying the learned techniques, possibly with the support of further courses and/or retreats. Four participants had not yet made plans and one participant wanted to follow a mindfulness trainer course. Besides, little system change was needed to integrate IMP in the existing infrastructure of our center as we have offered similar group interventions for many years.

#### Preliminary Effectiveness

Data of two consecutive training groups (*n* = 14 and *n* = 11) were gathered. *N* = 22 individuals in the control group were recruited. Mean age of the total sample at pre-training assessment was 49.6 years (SD, 10.7; range, 22–68), with *n* = 4 (8.5%) males (one in the training group and three in the control group). Mean age of the training participants (51.4 years; SD, 10.8) did not differ significantly from that of the individuals in the control group (47.5 years; SD, 10.4). Table 5 shows the results of the repeated measures ANOVA in the training group and the control group. On three of the six main measures, self-compassion, empathy and compassion fatigue, the IMP training had a significant effect in a positive direction, with partial eta squared values of 0.119, 0.109 and 0.104, respectively, meaning almost large effect sizes between medium and large. Also, on the FFMQ subscale ‘non-reactivity to inner experience’ and on the SCS subscale ‘isolation’, the training had a significantly positive effect (with partial eta squared values of 0.093 and 0.088, respectively, meaning medium to large effect sizes). The amount of time spent on home practice per week was not significantly correlated with the difference scores of the six measures (data not shown). One participant reported to have experienced adverse reactions, in the form of flashbacks and dreams. Four participants reported problems with awareness: ‘because of increased awareness of my own tendencies, I more often say “no” to everybody (implicitly or explicitly). I think this is good for me, but others do not always agree’, ‘being over-aware’, ‘more often than desirable (for me), being aware of everything’, ‘experiencing the difference that others do not have this awareness’. Table 6 presents the topics that participants had missed in the training.

**Table 5:** Results of the two-way mixed design ANOVA

Outcome measure	Group^a^	Pre-training assessment		Post-training assessment		Group effect/ANOVA group × time effect
		M	SD	M	SD	
Mindfulness (FFMQ)	Training	138.03	15.09	142.04	11.31	*F*(1, 44) = 1.983, *p =* .166, *ƞ*^2^ = .043
	Control	140.14	11.84	141.07	13.37	
Observing	Training	29.29	3.52	30.08	3.43	*F* = .365, *p =* .549, *ƞ*^2^ = .008
	Control	29.09	3.78	29.54	4.09	
Describing	Training	29.58	4.34	29.88	3.83	*F* = .051, *p =* .823, *ƞ*^2^ = .001
	Control	29.45	3.51	29.86	3.45	
Acting with awareness	Training	25.17	4.56	26.12	3.28	*F* = .876, *p =* .354, *ƞ*^2^ = .020
	Control	25.27	3.13	25.59	2.82	
Non-judging of inner experience	Training	30.40	5.47	30.83	4.28	*F* = .110, *p =* .742, *ƞ*^2^ = .002
	Control	31.09	4.68	31.14	5.17	
Non-reactivity to inner experience	Training	23.63	3.08	25.17	2.66	*F* = 4.422, *p =* .041, *ƞ*^2^ = .093*
	Control	25.23	3.45	25.02	3.37	
Self-compassion (SCS)	Training	81.54	8.82	85.35	8.41	*F*(1, 44) = 5.917, *p =* .019, *ƞ*^2^ = .119*
	Control	90.86	12.74	88.89	11.66	
Self-kindness	Training	13.64	1.91	14.56	1.98	*F* = .414, *p =* .523, *ƞ*^2^ = .009
	Control	14.71	2.92	15.00	2.20	
Self-judgement	Training	13.01	2.99	13.53	2.77	*F* = 2.861, *p =* .098, *ƞ*^2^ = .061
	Control	15.57	2.87	14.88	2.70	
Common humanity	Training	13.36	2.20	13.72	2.23	*F* = 1.493, *p =* .228, *ƞ*^2^ = .033
	Control	14.10	2.49	13.64	1.87	
Isolation	Training	14.24	2.15	14.77	2.55	*F* = 4.257, *p =* .045, *ƞ*^2^ = .088*
	Control	16.14	3.00	15.14	3.38	
Mindfulness	Training	13.96	1.27	14.88	1.64	*F* = 3.718, *p =* .060, *ƞ*^2^ = .078
	Control	15.33	2.67	15.23	2.27	
Over-identification	Training	13.32	2.23	13.88	1.99	*F* = .347, *p =* .559, *ƞ*^2^ = .008
	Control	15.00	3.03	15.00	2.47	
Empathy (EQ)	Training	45.47	8.52	47.94	9.02	*F*(1, 44) = 5.365, *p =* .025, *ƞ*^2^ = .109*
	Control	49.85	8.35	48.07	9.97	
Stress (PSS)	Training	14.28	4.85	12.76	3.30	*F*(1, 44) = 1.043, *p =* .313, *ƞ*^2^ = .023
	Control	12.43	5.67	12.50	4.85	
Compassion satisfaction (ProQOL)	Training	38.29	3.78	37.58	4.10	*F*(1, 44) = .111, *p =* .740, *ƞ*^2^ = .003
	Control	38.95	4.18	38.50	4.71	
Compassion fatigue (ProQOL)	Training	42.98	6.53	39.92	4.78	*F*(1, 44) = 5.114, *p =* .029, *ƞ*^2^ = .104*
	Control	40.36	5.65	40.27	6.68	

Effect sizes reported are partial eta squared

*M*, mean; *SD*, standard deviation; *FFMQ*, Five Facet Mindfulness Questionnaire; *SCS*, Self-Compassion Scale; *EQ*, Empathy Quotient; *PSS*, Perceived Stress Scale; *ProQOL*, Professional Quality of Life Scale

*Significant outcomes

**Table 6:** Topics that IMP training participants had missed

- I think that I still miss a link with the daily work practice. We are taught great things, but can we use them and how? Is it allowed at work? Does it fit in one’s work culture?	
- Silence meditation, but that is of course not interpersonal	
- Comeback session	
- Application (e.g. contemplations/themes) in caregiver’s profession	
- Application in personal situation	
- Maybe more variation in exercises, as I found the exercises in pairs sometimes dull and lengthy	

## Discussion

This study aimed to evaluate the feasibility of implementing an IMP training for health care professionals as well as to assess its preliminary effectiveness on levels of mindfulness, self-compassion, empathy and professional quality of life compared with a control group. The IMP training showed high feasibility on the assessed domains. There were no dropouts. Participants were very satisfied with the content of the training, the teachers, the course material and the organisation. Preliminary effects of the IMP training were significant improvements regarding levels of self-compassion, empathy and compassion fatigue. No significant effects on levels of mindfulness, stress and compassion satisfaction were observed. Subscale analyses showed improvements in non-reactivity to inner experience (pertaining to the mindfulness scale FFMQ), and in isolation (pertaining to the self-compassion scale SCS). Mild adverse reactions were reported by five participants.

In this study, we did not further explore effects of the training on burnout as part of the Compassion Fatigue subscale of the ProQOL. First, burnout showed a poor internal consistency in our sample. Second, the concept of burnout is not yet well defined. A systematic review by Rotenstein et al. (2018) demonstrated that burnout among physicians show substantial variability in prevalence estimates, besides marked variation in definitions and assessment methods. Besides, in their editorial, Schwenk and Gold (2018) questioned the way in which burnout is assessed and commented on the fact that physicians who report burnout—based on one or two questions—already receive recommendations for treatments before there is an actual understanding of the diagnosis. However, O’Connor et al. (2018) identified eight validated burnout measures in their literature review on burnout in mental health professionals and found high rates of burnout among mental health professionals, with an estimated prevalence of emotional exhaustion of 40%.

The concept of empathy and compassion-fatigue has been studied more in health care workers in general than in *mental* health care staff. Burnout, compassion-fatigue and compassion-satisfaction were studied among staff in community-based mental health services by Rossi et al. (2012), using the ProQOL, yet in this study only associations were explored but no intervention. It should be mentioned that Ricard (2015), an experienced meditator, supported by neuroscientific findings presented by Klimecki et al. (2014), suggested that ‘compassion fatigue’ is a misnomer. Klimecki and Singer (2011) proposed to replace the term compassion fatigue by the term ‘empathic distress fatigue’ and Ricard (2015) argued it would be better to speak of ‘empathy fatigue’ because it is associated with fatigue, whilst compassion is associated with positive emotions. Empathically feeling into another’s suffering leads to distress. Compassionate commitment to relieve suffering, including one’s own, has soothing and restorative effects. It should be mentioned that although the concepts of ‘empathy’ and ‘compassion’ may resemble each another, there are differences. Likewise, Baron-Cohen and Wheelwright (2004) observed four varieties of empathy: (1) the feeling in the observer must match that of the person observed (e.g. you feel fright when you see someone else’s fear), (2) the feeling in the observer is simply appropriate to the other person’s emotional state in some other way, even though it does not exactly match it (e.g. you may feel pity at someone else’s sadness, (3) the feeling in the observer may be any emotional response to another’s emotion (e.g. an observer feeling pleasure at another’s pain). This is referred to as ‘contrast empathy’ and (4) the feeling in the observer must be one of concern or compassion with another’s distress. Only the latter definition shows some overlap with compassion. The concepts of empathy and self-compassion in relation to interpersonal mindfulness training among mental health care workers are seemingly not studied to date.

We argue that some of the training effects may have been less obvious due to a possible ‘ceiling effect’ for level of mindfulness, as—on top of the minimum requirement of a previously followed MBSR or MBCT course—a considerable number of the study participants appeared to be experienced mindfulness practitioners, of whom some also had previous experience with Insight Dialogue, a Buddhist communication programme which is the source and inspiration of the secular IM. The amount of time spent in formal practice between sessions was not associated with outcome. However, there could well have been an effect of *informal* practice, because participants could have been applying what they had learned—intentionally and/or unconsciously—in their everyday interpersonal communications.

### Limitations and Future Research

A limitation that should be mentioned is the small sample size of our study. Post hoc analysis in G*Power (Faul et al. 2009), based on the observed significant outcomes for self-compassion, empathy and compassion fatigue, revealed a power of 0.70. Another limitation is that our study may have been limited by common method bias given that we only used self-rating scales and no external validation measures. This may have led to spurious effects due to the measurement instruments rather than to the constructs being measured (Podsakoff et al. 2003). Furthermore, the vast majority of the total sample were female, with only one male in the training group and three males in the control group. Therefore, we could not analyse gender differences. Indeed, our figures concur with the overrepresentation of women in the health workforce (World Health Organization 2008). Finally, we examined *self*-compassion, but it is important to examine changes in *compassion for others* as well. First, compassion and self-compassion were shown to be not significantly related (Lopez et al. 2018). Second, the ProQOL measures compassion satisfaction and compassion fatigue.

Future studies might examine possible mechanisms through which the IMP works in some areas. Importantly, the adverse reactions reported here deserve attention in future research. In addition, the concept of burnout could be examined further.
